# A reliable technique for karyotyping mouse oocytes prepared by a gradual fixation/air-drying method followed by multicolour FISH

**DOI:** 10.1242/bio.060188

**Published:** 2023-12-13

**Authors:** Toshiaki Hino, Hirokazu Kusakabe

**Affiliations:** Department of Biological Sciences, Asahikawa Medical University, Midorigaoka-Higashi 2-1-1-1, Asahikawa 078–8510, Japan

**Keywords:** Meiosis, Karyotype, Chromosome, Segregation error, Mice, Multicolour FISH

## Abstract

Chromosome segregation errors during oocyte meiosis increase with age and lead to aneuploidy; hence, the mechanism has been studied extensively. The mouse is the most widely used experimental animal for this purpose. However, the lack of a reliable and efficient technique for karyotyping mouse oocytes has limited comprehensive studies of chromosome-specific segregation errors in this animal model. Here, we developed a novel karyotyping technique for mouse oocytes by applying multicolour fluorescence *in situ* hybridisation (FISH) to chromosome slides prepared by a gradual fixation/air-drying method, which is best suited to avoid rupture of oocyte membrane and artificial loss of chromosomes. The success rate of karyotyping meiosis I and II oocytes was about 30%, which improved to over 90% when the oocytes were ‘flattened’ during fixation and the chromosome specimens were denatured at 4°C. When this technique was applied to the karyotyping of meiosis II oocytes from aged female mice and from young female mice injected with colchicine, more than 80% of the oocytes were successfully karyotyped and the number of chromosomes was identified on all aberrant chromosomes. In conclusion, our technique allows for the efficient and reliable karyotyping of mouse oocytes.

## Teaser

**Summary:** Mouse oocytes can be karyotyped efficiently and reliably by applying multicolour FISH to chromosome slides prepared by a gradual fixation/air-drying method best suited to avoid the artificial loss of chromosomes.

## INTRODUCTION

Meiosis is a special cell division that occurs during oocyte and spermatozoa production, in which two divisions occur successively. In the first division (meiosis I, MI), homologous chromosomes are equally separated into the two daughter cells. In the second division (meiosis II, MII), sister chromatids are separated equally into the next two daughter cells. Chromosome segregation errors occurring in meiotic stages that increase with age will produce oocytes with a numerical abnormality in chromosome constitution (Hassold and Hunt, 2001; Hassold et al., 2007; Nagaoka et al., 2012; Franasiak et al., 2014). Involvement of such abnormal oocytes in the fertilization induces aneuploid zygotes, resulting in early abortion and developmental defects in the fetus. Much research has been devoted to understanding the mechanisms of chromosome segregation errors in oocyte meiosis. The mouse is one of the most widely used laboratory animals to study meiosis. The various methods for chromosome research and the existence of many genetically engineered mice have contributed significantly to our understanding of meiosis. However, because a reliable method for identifying and analysing individual chromosomes (i.e. karyotype analysis) in meiotic oocytes has not yet been established, little progress has been made in studying chromosome-specific segregation errors, such as whether the frequency of segregation errors varies among chromosomes and, if so, what molecular mechanism underlies this phenomena.

In this study, we developed a reliable method for karyotyping oocytes to identify individual chromosomes with meiotic segregation errors by combining multicolour fluorescence *in situ* hybridisation (FISH) with chromosome preparations made by the gradual fixation/air-drying method (Mikamo and Kamiguchi, 1983). We validated this method by applying it to the cytogenetic analysis of oocytes from aged female mice and from young female mice treated with an aneugen, colchicine. Multicolour FISH is characterised by using specific fluorescent DNA probes for individual chromosomes to colour-code and identify all chromosomes in one hybridisation (Liehr and Kosyakova, 2017). Multicolour FISH karyotype analysis is widely used as a cytogenetic assay to detect structural and numerical aberrations of the chromosomes in somatic cells. Gradual fixation/air-drying is a method to make oocyte chromosome preparations (Mikamo and Kamiguchi, 1983), which is characterised by the gradual fixation of oocytes using three different fixative solutions in stages to prevent the rupture of the oocyte membrane and the artificial loss of chromosomes. Thus, the method generates highly reliable and reproducible chromosome preparations.

## RESULTS AND DISCUSSION

Table 1 shows the number of oocytes that were successfully karyotyped when chromosome slides made by the gradual fixation/air-drying method were hybridised with multicolour FISH probes with or without technical modifications. The oocytes were obtained from young (2-month-old) female mice. The success rate of the karyotype analysis without technical modifications was very low. In MI oocytes, only 10 (27%) of the 37 oocytes collected were successfully karyotyped by multicolour FISH. Only 10 (34%) of the 29 oocytes collected in MII oocytes were successfully karyotyped by multicolour FISH. Most MI and MII oocytes that failed to be karyotyped showed poor-quality images of chromosomes with uneven fluorescence caused by oocyte wrinkles and strong background fluorescence in the ooplasm, both of which overlapped with the chromosomes (Fig. S1A). In these cases, the karyotype analysis failed because it was impossible to determine whether the chromosome was positive or negative for the multicolour FISH probes.

**Table 1.**
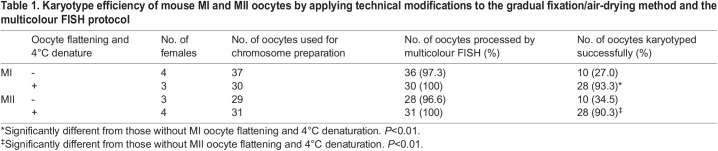
Karyotype efficiency of mouse MI and MII oocytes by applying technical modifications to the gradual fixation/air-drying method and the multicolour FISH protocol

**Fig. 1.**
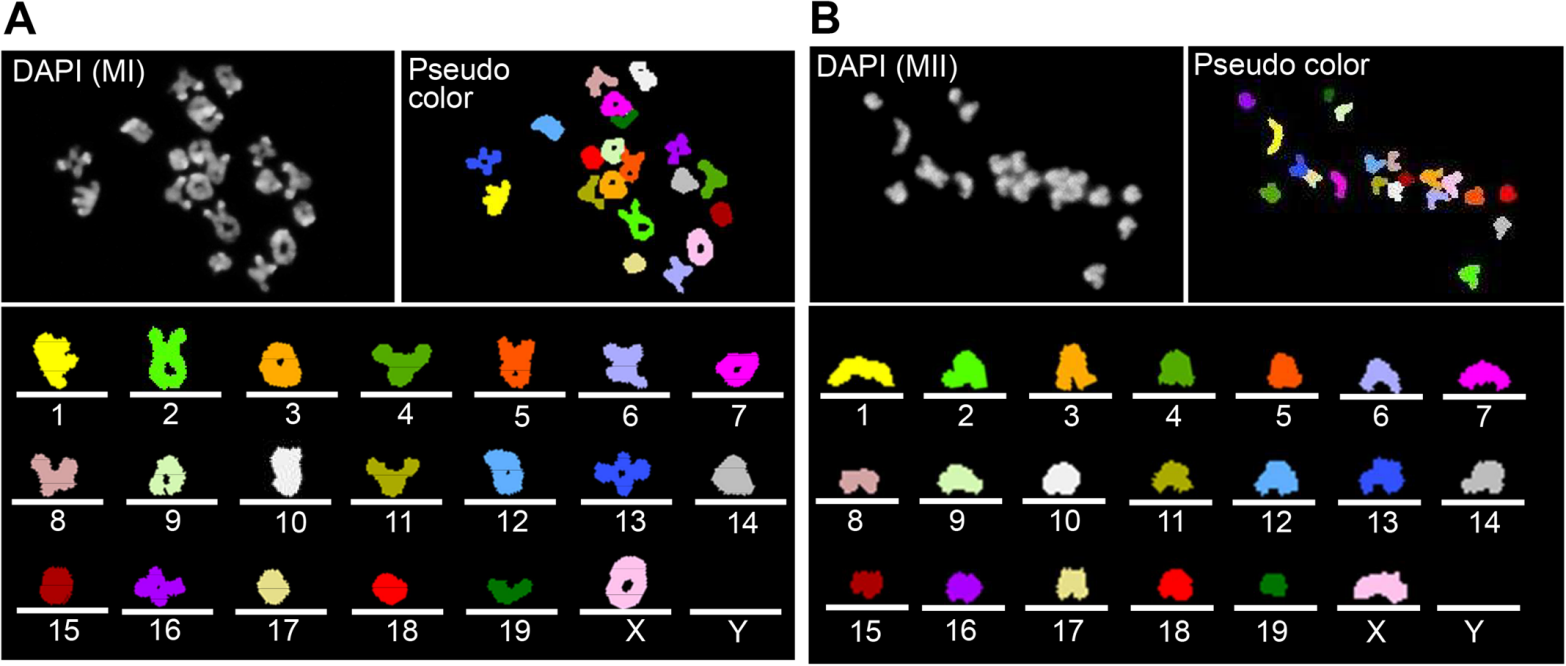
**Karyogram of MI and MII oocytes.** (A,B) Representative image of karyotyping of an MI oocyte (A) and an MII oocyte (B). Note that a mouse oocyte comprises 19 autosomes and an X chromosome (sex chromosome). All autosomes were arranged by number on idiograms, with the sex chromosome placed last.

Because oocytes are larger and thicker than somatic cells, the non-specific fluorescence caused by the cytoplasm becomes stronger. Furthermore, in the denaturation step of chromosomal DNA, oocytes were found to swell during alkaline treatment of the chromosome slides with sodium hydroxide (NaOH) at room temperature (Fig. S2). Such swelling of the oocytes will cause the formation of wrinkles of the oocyte membrane during dehydration and drying processes after denaturation. To overcome the problems, the oocytes were flattened and thinned during oocyte fixation (see the Materials and Methods). The denaturing of chromosome slides with NaOH was performed at 4°C instead of room temperature to avoid oocyte swelling. As a result of these two technical improvements, oocyte wrinkles and non-specific fluorescence disappeared in almost all oocytes (Fig. S1B) and the success rate of the karyotype analysis (Fig. 1) improved significantly to more than 90% of MI and MII oocytes (Table 1, *P*<0.01).

**Fig. 2.**
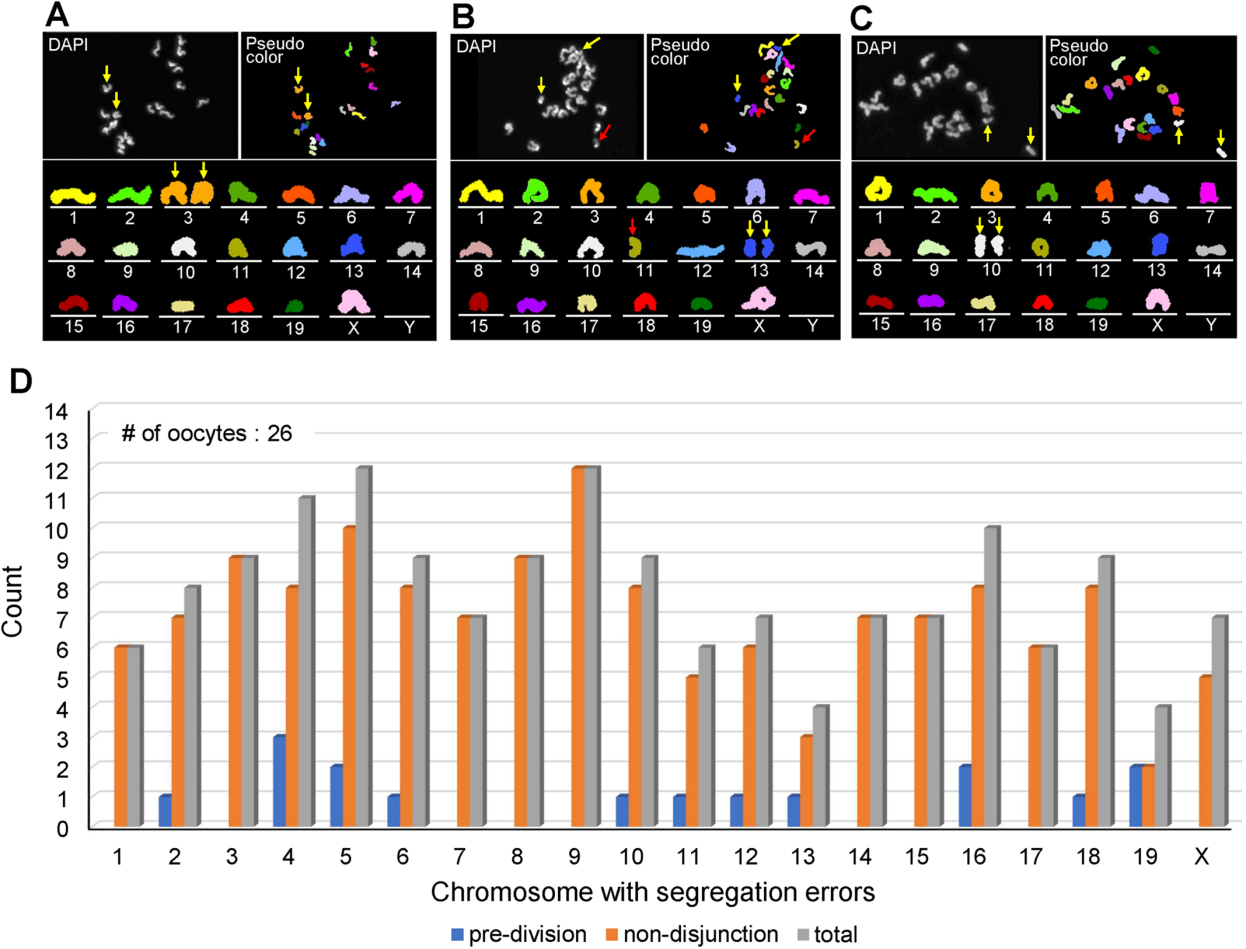
**Karyotype analysis of MII oocytes obtained from young female mice injected with colchicine and from aged female mice.** (A,B) Karyotypes of the MII oocytes obtained from colchicine-injected female mice. (A) The karyotype in which the non-disjunction (arrows) of chromosome 3 was detected. (B) Karyotype in which both balanced pre-division of chromosome 13 (yellow arrows) and unbalanced pre-division of chromosome 11 (red arrow) were detected. (C) The karyotype of the MII oocyte was obtained from aged female mice. The balanced pre-division of chromosome 10 was detected. (D) Frequency of aberrations in each chromosome of MII oocytes with aberrations in the chromosome in the colchicine group. This result indicates that segregation errors occur on any chromosome.

We validated our karyotyping method by applying it to the chromosome analysis of mouse MII oocytes from young female mice injected intraperitoneally with colchicine. Colchicine injection into female mice at the time of human chorionic gonadotropin (hCG) injection is known to induce chromosome segregation errors in MI due to inhibition of microtubule formation (Mailhes and Yuan, 1987), resulting in non-disjunction (i.e. extra or missing univalent chromosomes), balanced pre-division (i.e. separated chromatids for one univalent chromosome) and unbalanced pre-division (i.e. extra or missing chromatids) in MII oocytes. However, it remains to be determined whether segregation errors occur on a specific chromosome. Of the 50 oocytes obtained from colchicine-injected female mice, multicolour FISH processed 46 (92%) oocytes and 43 (86%) of these were successfully karyotyped (Table 2). Twenty-six (63%) of the 43 karyotyped oocytes showed aberrations of the chromosome, which was significantly higher than the 0% of the control group (*P*<0.01). The most common type of chromosome aberrations observed was non-disjunction (Fig. 2A). Balanced and unbalanced pre-divisions were also observed in some oocytes (Fig. 2B). The contribution of each of the 1 to 19 autosomes and the X chromosome to those segregation errors is summarised in Fig. 2D, indicating that colchicine-induced chromosome segregation errors occur in any chromosome, not in a specific chromosome.

**Table 2.**
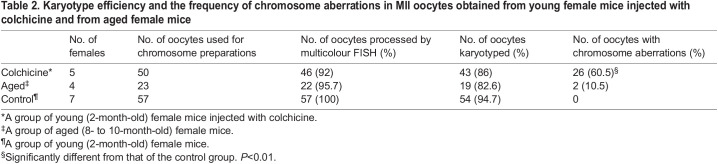
Karyotype efficiency and the frequency of chromosome aberrations in MII oocytes obtained from young female mice injected with colchicine and from aged female mice

We also investigated whether our method could be applied to the karyotyping of MII oocytes obtained from aged (8- to 10-month-old) female mice. Twenty-three oocytes were obtained from four female mice, of which 19 (83%) were successfully karyotyped by multicolour FISH (Table 2). Balanced pre-division of sister chromatids was detected in two (11%) of the 19 oocytes: one observed on chromosome 10 (Fig. 2C) and the other on chromosome 13. Although the number of oocytes examined in the aged group was small, together with the data obtained from the colchicine-injected female mice, the present results show that our karyotyping method can efficiently contribute to a comprehensive study of chromosome segregation errors focusing on individual chromosomes. Furthermore, not a single hypohaploid appeared among the 54 oocytes in the control group, proving that there was no artificial loss of chromosomes due to rupture of the oocyte membrane and that the chromosome slides prepared by our method are highly reliable.

Tarkowski's method (Tarkowski, 1966) is widely used for cytogenetic studies of mammalian oocytes, such as mouse and human oocytes. This method uses a single fixative and has the advantage of being simple and easy to learn but has the disadvantage of damaging the oocyte membrane during fixation (Sugawara and Mikamo, 1986), which causes artificial loss of chromosomes. On the other hand, the gradual fixation/air-drying method developed in our laboratory (Mikamo and Kamiguchi, 1983), which requires considerable time and training to master, is highly reliable for preparing chromosome slides because the stepwise gentle fixation of the oocyte with three different fixatives preserves the oocyte membrane (Sugawara and Mikamo, 1986), and thus the artificial loss of chromosomes does not occur.

In addition to multicolour FISH, chromosome banding and genome-based methods such as comparative genomic hybridisation (CGH) and next-generation sequencing (NGS), respectively, have been used for karyotype analysis. Chromosome banding is a method in which chromosome preparations are stained with quinacrine mustard (Q-banding) or Giemsa's solution after treatment of chromosome preparations with proteolytic enzymes (G-banding) to identify individual chromosomes based on the band patterns specific to each chromosome. However, this method is inadequate for the karyotype analysis of oocyte chromosomes because they are extremely condensed compared to those of somatic cells; therefore, the bands must appear sufficiently to identify the chromosomes. CGH- and NGS-based karyotyping are characterised by detecting chromosomal aberrations based on copy number changes in chromosomal DNA (Gutiérrez-Mateo et al., 2011; Fiorentino et al., 2014). In mice, NGS is successfully applied as a tool for karyotype analysis to detect aneuploidy at the level of single chromosomes in mouse oocytes (Treff et al., 2016). However, NGS-based karyotyping and CGH for oocytes cannot detect balanced pre-division, which is not reflected in DNA copy number changes. Balanced pre-division is the most common type of chromosome segregation error that increases with age in mouse oocytes (Sakakibara et al., 2015). The inability to detect this type of error makes these methods inadequate for karyotype analysis in oocytes. Therefore, a multicolour FISH using chromosome preparations made by the gradual fixation/air-drying method is currently the only method for detailed and convincing analysis of chromosome aberrations in mouse oocytes.

In conclusion, we have successfully developed a reliable karyotyping method for mouse oocytes. Our technique allows the chromosome-specific identification of any chromosome segregation errors in oocytes and will contribute to providing new findings in reproductive science and medicine to improve our understanding of how age, environmental factors and disease states affect oocyte aneuploidy. It could also be used to evaluate the efficacy of therapeutic interventions to reduce the incidence of age-related oocyte aneuploidy.

## MATERIALS AND METHODS

### Animals

Six-week-old female ICR mice were purchased from SLC Inc. (Shizuoka, Japan) and maintained under specific pathogen-free conditions with controlled light, temperature and humidity (light between 7:00 and 19:00, 22±2°C, 50±10% humidity) for at least 2 weeks before performing the experiments. Some were housed until they were 8 to 10 months old. All animal experiments were conducted according to the Asahikawa Medical University Animal Experiments Guidelines.

### Chemicals

Unless otherwise stated, the chemicals were purchased from Nacalai Tesuque (Kyoto, Japan).

### Collection of MI and MII oocytes

The MI oocytes were collected as follows. Female mice were injected intraperitoneally with equine chorionic gonadotropin (eCG; ASKA Pharmaceutical, Tokyo, Japan), and 46 h later, MI oocytes in the germinal vesicle stage were collected from large antral follicles and transferred to Toyoda–Yokoyama–Hosi (TYH) medium (Toyoda et al., 1971). After the cumulus cells surrounding the oocytes were removed by vigorous pipetting, the oocytes were transferred to a TYH medium containing 0.02 µg/ml vinblastine sulfate. The oocytes were microscopically examined at 30-min intervals and oocytes 1.5 to 3 h after the disappearance of the nuclear membrane were used to prepare chromosome slides.

The MII oocytes were collected as follows. Female mice were injected intraperitoneally with eCG, followed 48 h later by hCG injection (ASKA Pharmaceutical). In some female mice, 0.3 mg/kg colchicine (diluted in saline) was injected intraperitoneally at the time of hCG injection. Fifteen hours after hCG injection, a clot of cumulus cells and oocytes was collected from the ampulla and transferred to a TYH medium containing 0.02% hyaluronidase to disperse the cumulus cells. After the cumulus cells were removed, the oocytes were washed three times with TYH medium and used to prepare chromosome slides.

### Preparation of chromosome slides and analysis of oocytes by multicolour FISH

The diagram of the chromosome slide preparation procedure is shown in Fig. S3. Oocytes were incubated in phosphate-buffered saline containing 0.5% actinase E (Kaken Pharmaceutical Co., Shizuoka, Japan) at 37°C to digest their zona pellucida so as not to interfere with oocyte swelling during the subsequent hypotonic treatment. The zona-free oocytes were transferred to TYH medium, washed three times and then treated with a hypotonic solution (1:1 mixture of 1.2% sodium citrate and 18% foetal bovine serum) for 10 min at room temperature. Oocyte chromosome slides were prepared according to Mikamo and Kamiguchi (1983). The oocytes were transferred to fixative I (5:1:4 mixture of methanol, acetic acid and distilled water) and treated until they began to turn transparent. Next, the oocytes were placed on the slide with a small amount of fixative I and then covered with fixative II (3:1 mixture of methanol and acetic acid) by slowly releasing fixative II on the slide, a short distance from the oocytes, as shown in Fig. S4A. The slide was placed in a Coplin jar containing fixative II for 2 min and then in another Coplin jar containing fixative III (3:3:1 mixture of methanol, acetic acid and distilled water) for 1 min. The slide was air-dried at 22–24°C under 50–60% humidity conditions.

In some experiments, the slide was left in place for approximately 20 s before being transferred to Coplin jars containing fixative II. During this time, the level of fixative II on the slide was lowered gradually by evaporation, and the oocytes flattened and reduced in thickness by being pushed down by the drop in liquid level (see Fig. S4).

For multicolour FISH analysis, chromosome slides were hybridised with a multicolour FISH probe (21xMouse; MetaSystems Probes, Altlußheim, Germany) according to the manufacturer's protocol for somatic cells with some modifications where appropriate. All sodium chloride–sodium citrate (SSC) solutions used as hybridisation buffer were prepared from 20× SSC. Chromosome slides were incubated in 2× SSC at 70°C for 30 min and washed in 0.1× SSC for 1 min at room temperature. Chromosome slides were then denatured in 0.07 M NaOH for 1 min at room temperature. In some experiments, the temperature of the 0.07 M NaOH was set at 4°C instead of room temperature. After denaturation, the slides were washed in 0.1× SSC and 2× SSC for 1 min at 4°C, and dehydrated in 70%, 95% and 100% ethanol and air-dried. A multicolour FISH probe was incubated at 75°C for 5 min, followed by incubation at 37°C for 30 min, and was applied to the slides. Hybridisation proceeded in a moist chamber at 37°C for 48 h. After hybridisation, the slides were washed by incubation in 0.4× SSC at 72°C for 2 min, followed by 2× SSC with 0.05% Tween20 at room temperature for 30 s. The slides were then rinsed in distilled water, air-dried and cover-slipped with 4′,6-diamidino-2-phenylindole (DAPI)/Antifade (MetaSystems Probes) for counterstaining.

The images of five fluorescent colours (aqua, green, orange, red and near-infrared) and DAPI of the chromosome spreads were acquired using a fluorescence microscope (BX51; Olympus, Tokyo, Japan) with a high-sensitivity digital camera (α7 s; Sony, Tokyo, Japan). All images were imported into ChromaWizard software (Auer et al., 2018), and karyotype analysis was performed in the ChromaWizard software.

### Statistical analysis

The percentage data were analysed using Fisher's exact test.

## Supplementary Material

10.1242/biolopen.060188_sup1 Supplementary information

## Diversity and inclusion statement

We are committed to fostering diversity, equity, and inclusion in the field of science. We believe that a diverse and inclusive research community is essential for scientific progress and that it is also a moral imperative. We recognise the historical under-representation and marginalization of women, ethnic and sexual minorities, and individuals with disabilities in our field. Our authorship team reflects a commitment to diversity.
