# Supplementary material for: A reliable technique for karyotyping mouse oocytes prepared by a gradual fixation/air-drying method followed by multicolour FISH
Source: Biol Open. 2023 Dec 13;12(12):bio060188. doi: 10.1242/bio.060188 (PMC10732245; doi:10.1242/bio.060188)
Supplement: Supplementary information [file biolopen-12-060188-s1.pdf]

Fig. S1

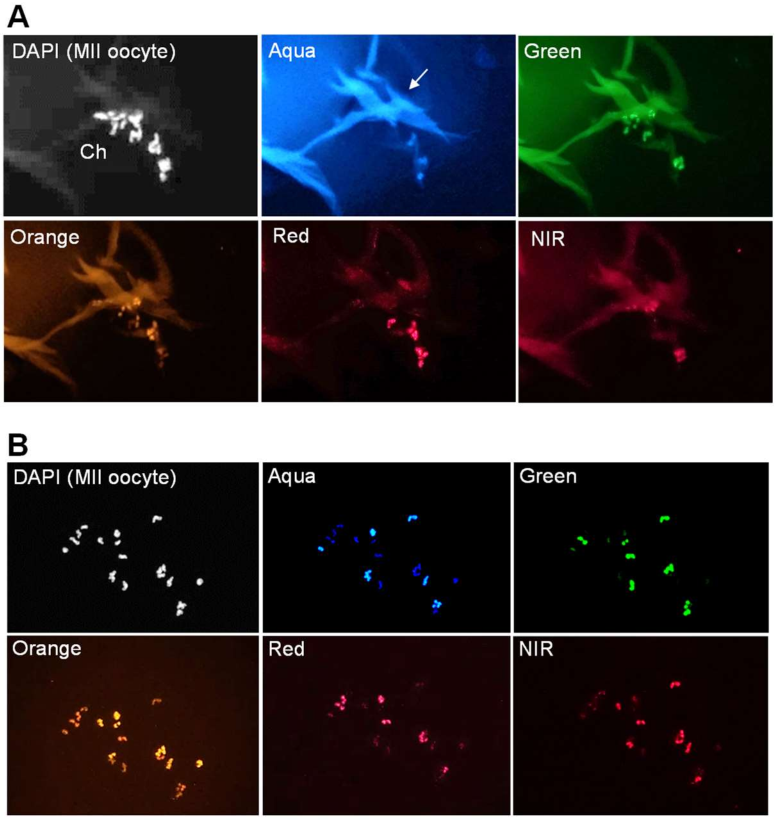

Fig. S1. Fluorescence images of the MII oocyte chromosomes made by the gradual fixation/air-drying method followed by multicolour FISH. (A) Fluorescence images of an MII oocyte without the application of technical modifications. The uneven fluorescence caused by oocyte wrinkles and the strong background fluorescence in the cytoplasm overlapped with the fluorescence of the chromosomes (Ch). Note that this is evident in aqua, where the fluorescence of chromosomes with overlapping wrinkles (arrow) is barely discernible. (B) Fluorescence images of an MII oocyte with technical modifications. Background fluorescence was significantly attenuated, allowing karyotype analysis based on chromosome fluorescence.

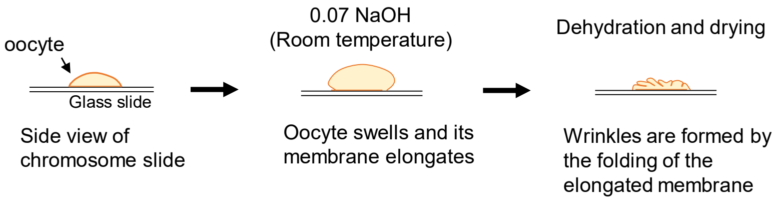

Fig. S2. Formation of oocyte wrinkles during denaturation, dehydration, and drying of chromosome slides. Oocytes on glass slides swell and their membrane elongates during denaturation (NaOH treatment). The elongates membrane is folded during dehydration and drying, resulting in oocyte wrinkles.

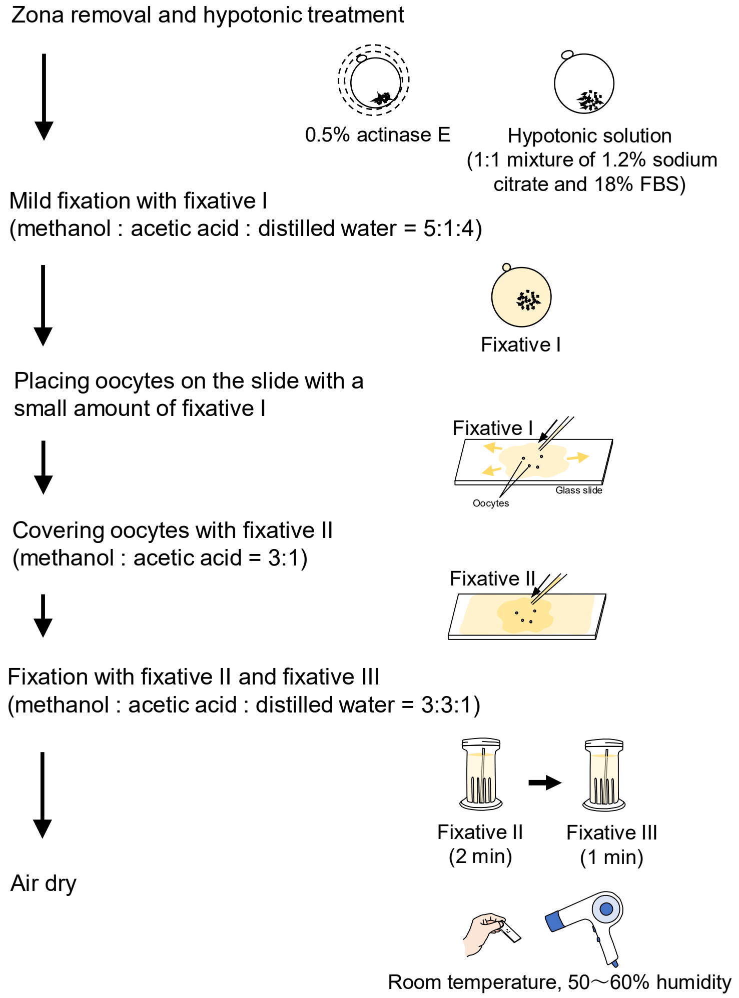

Fig. S3. Diagram of the procedure of chromosome slide preparation. For a detailed description of the procedure, see Materials and Methods section.

Biology Open • Supplementary information

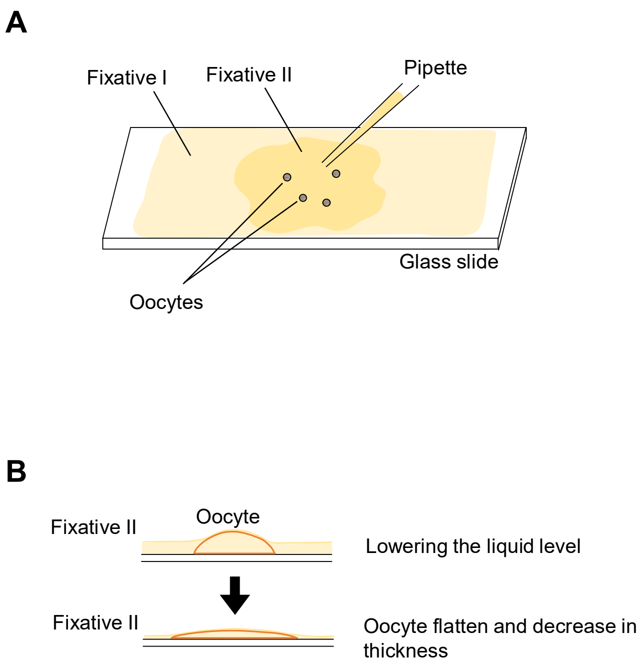

Fig. S4. Reduction of oocyte thickness by flattening. (A) Diagram of the oocytes and the glass slide after the oocytes were placed on a glass slide together with fixative I and a small amount of fixative II was poured onto the glass slide. (B) Side view of the oocyte in (A). To reduce the thickness of the oocytes, the glass slide was left in place for approximately 20 s before being placed in the Coplin jar containing fixative II. During these 20 s, the level of the fixative II covering the oocytes gradually dropped, and the oocytes were pushed down, flattened, and reduced in thickness by the drop in liquid level.

Biology Open • Supplementary information
